# Relación entre actividad física, sedentarismo y obesidad en adultos, Colombia, 2015

**DOI:** 10.7705/biomedica.7014

**Published:** 2023-12-29

**Authors:** Ana Yibby Forero, Gina Emely Morales, Luis Carlos Forero

**Affiliations:** 1 Grupo de Nutrición, Subdirección de Investigación Científica y Tecnológica, Dirección de Investigación en Salud Pública, Instituto Nacional de Salud, Bogotá, D.C., Colombia Instituto Nacional de Salud Bogotá D.C Colombia

**Keywords:** adulto, obesidad, ejercicio físico, conducta sedentaria, salud pública, enfermedades no transmisibles, Adult, obesity, exercise, sedentary behavior, public health, noncommunicable diseases

## Abstract

**Introducción.:**

La inactividad física y los comportamientos sedentarios demostraron ser factores de riesgo en la prevalencia de enfermedades como la obesidad.

**Objetivo.:**

Analizar la relación entre la actividad física, el sedentarismo y el estado nutricional en la población de 18 a 64 años en Colombia, 2015.

**Materiales y métodos.:**

Se llevó a cabo el análisis secundario de la encuesta de nutrición de Colombia en 2015, utilizando variables sociodemográficas (edad, sexo, etnia, área geográfica, región e índice de riqueza) y, además, peso, talla, actividad física y sedentarismo. Se estimaron proporciones e intervalos de confianza al 95 %, utilizando la prueba de ji al cuadrado, regresión logística y razón de momios *(Odds ratio).*

**Resultados.:**

La población analizada incluyó 16.607 individuos (54,8 % mujeres y 45,2 % hombres) de 18 a 64 años. Los hombres tuvieron más sobrepeso (37,6 %) que las mujeres, mientras que la obesidad fue más frecuente en mujeres (22,1 %). El 48,3 % no cumple con la recomendación de actividad física; 56,9 % de los adultos pasa dos o más horas al día mirando pantallas. Hubo una asociación significativa (p<0,050) entre el incumplimiento de las recomendaciones de actividad física y la obesidad. Además, el cumplir con la recomendación de actividad física disminuye 1,25 veces la probabilidad de obesidad.

**Conclusión.:**

La obesidad es un problema de salud para la población adulta, con mayor prevalencia en las mujeres y en los mayores de 30 años. Se relaciona con el incumplimiento de las recomendaciones sobre actividad física y con los comportamientos sedentarios (los cuales son más prevalentes en los afrodescendientes), el cuartil alto de riqueza, el vivir en área urbana y el vivir en Bogotá.

## Abstract (en)

**Introduction.:**

Physical inactivity and sedentary behaviors were shown to be risk factors in the prevalence of diseases such as obesity.

**Objective.:**

To conduct an analysis of the relationship between physical activity, sedentary lifestyle and nutritional status in the population aged 18 to 64 years in Colombia, 2015.

**Materials and methods.:**

A secondary analysis of the 2015 Colombian nutrition survey was done, using sociodemographic variables (age, sex, ethnicity, geographic area, region, wealth index), along with weight, height, physical activity and sedentary lifestyle. Proportions and 95% confidence intervals, chi-square, logistic regression and odds ratio were estimated.

**Results.:**

The analyzed population included 16,607 individuals (54.8% women and 45.2% men) aged 18 to 64 years. Men were more overweight (37.6%) than women, while obesity was more frequent in women (22.1%). It was observed that 48.3% did not meet the physical activity recommendations; 56.9% of adults spent two or more hours a day watching screens. There was a significant association (p<0.050) between non-compliance with physical activity recommendations and obesity. In addition, complying with the physical activity recommendation decreases the probability of obesity by 1.25 times.

**Conclusion.:**

Obesity is a health problem for the adult population, with a higher prevalence in women and in those over 30 years old. It is related to non-compliance with the recommendations about physical activity and sedentary behaviors (which are more prevalent in Afro-descendants), high wealth quartile status, and to reside in urban areas and to live in the Bogotá area.

Las enfermedades crónicas no transmisibles están afectando a los países, y la carga asociada de muerte y enfermedad está muy concentrada en aquellos de bajos y medianos ingresos. El detrimento de la productividad debido a las muertes prematuras y los costos, tanto de los individuos como de las naciones, al abordar estas enfermedades se consideran importantes obstáculos en la disminución de la pobreza y el desarrollo sostenible de los seres humanos ^1^.

Según la Organización Mundial de la Salud (OMS), en el 2017, más de 40 millones de individuos mueren cada año por enfermedades crónicas no transmisibles y el 80 % de estos fallecimientos son causados por aquellas que afectan el corazón o los vasos sanguíneos (17,7 millones anualmente), seguidas por el cáncer (8,8 millones), las enfermedades respiratorias (3,9 millones) y la diabetes (1,6 millones) ^2^.

Se ha demostrado que el realizar actividad física de forma regular y con una duración e intensidad suficientes, puede ayudar a prevenir y tratar las enfermedades crónicas no transmisibles; además, la actividad física tiene algunos efectos en la salud mental, retarda la manifestación de la demencia y ayuda al mantenimiento del peso saludable ^3^^,^^4^. En el 2018, en el estudio de Guthold *et al.*^5^, se estimó que el 27,5 % de las personas en el mundo son inactivas y que el porcentaje de inactividad en las mujeres (31,7 %) es mayor que en los hombres (23,4 %).

Las regiones con mayor prevalencia de inactividad física son Latinoamérica y el Caribe (39,1 %), seguidas por los países occidentales (36,8 %) y los de Asia con alto ingreso económico (35,7 %). Al analizar el alto porcentaje de inactividad física en Latinoamérica (43,7 %, las mujeres y 34,3 %, los hombres), se observó que Argentina, Brasil y Colombia contribuyeron con las más altas prevalencias ^5^.

Diversos estudios observacionales han demostrado que la falta de actividad física aumenta los riesgos de diferentes enfermedades no transmisibles, como la diabetes de tipo 2, las enfermedades cardiovasculares, los accidentes cerebrovasculares, el cáncer (de colon y mama) y la mortalidad prematura ^6^. En la actualidad, los comportamientos sedentarios son muy prevalentes, y la evidencia acumulada sugiere que se asocian con un mayor riesgo de enfermedad metabólica y mortalidad ^7^.

Entre los muchos comportamientos sedentarios, el tiempo frente a la pantalla entre los adultos representa un problema continuo y creciente en relación con los comportamientos y los resultados de salud. Los períodos prolongados de uso de la pantalla (interacción con dispositivos electrónicos que principalmente entregan contenido por medio de pantallas: incluye ver la televisión y el uso de celulares, tabletas o computadores), se han asociado con múltiples resultados adversos para la salud, como obesidad, diabetes de tipo 2, enfermedades cardiovasculares, alteraciones del sueño ^8^, problemas de salud mental como depresión ^9^ y mortalidad temprana en adultos ^7^^,^^10^^,^^11^.

La obesidad se ha vuelto cada vez más común a nivel global; casi el 40 % de los adultos tienen sobrepeso y del 10 al 15 % son obesos ^12^. En Colombia, desde la Encuesta Nacional de la Situación Nutricional de Colombia (ENSIN 2005), las prevalencias de la suma de sobrepeso y obesidad de la población adulta se han incrementado en 10,6 puntos porcentuales, pasando del 45,9 % en el 2005 al 56,5 % en el 2015, en forma similar a lo que es la tendencia mundial ^13^.

La obesidad, la inactividad física y el tiempo prolongado del uso de pantallas han demostrado ser factores de riesgo en la prevalencia de algunas enfermedades metabólicas entre las que se incluye el exceso de peso (sobrepeso y obesidad). Teniendo en cuenta lo anterior, se propuso un análisis de la relación entre la actividad física, el sedentarismo y el estado nutricional en la población de 18 a 64 años, utilizando la base de datos de la ENSIN Colombia 2015.

## Materiales y métodos

Se hizo un análisis secundario de los datos obtenidos en la ENSIN 2015 ^13^, la cual es una encuesta poblacional de corte transversal, en la que se utilizó un muestreo probabilístico y polietápico por conglomerados, con cobertura y representatividad nacional, en las áreas urbana y rural, para seis regiones del país y para las mediciones antropométricas desagregadas en 14 subregiones y 32 departamentos del país.

Para este estudio, se utilizó la información de antropometría, actividad física y comportamientos sedentarios en adultos entre los 18 y los 64 años de edad, correspondiente a 16.607 registros que tenían resultado para todas las variables seleccionadas.

Los indicadores del estado nutricional se definieron mediante mediciones corporales (peso y talla) y, a partir de estas, se determinó el índice de masa corporal (IMC) y se estableció la clasificación antropométrica teniendo en cuenta los puntos de corte de la OMS, adoptados en la Resolución 2465 de 2016 del Ministerio de Salud y Protección Social: IMC <18,5 corresponde a delgadez; IMC entre >18,5 y <25, a lo normal; IMC entre ≥25 y <30, a sobrepeso, e IMC ≥30, a obesidad ^14^.

La actividad física se categorizó según el cumplimiento o el incumplimiento de las recomendaciones de la OMS ^15^, como la realización, al menos, de 150 minutos a la semana de actividad física aeróbica con intensidad moderada, o 75 minutos semanales de actividad física aeróbica vigorosa, o una combinación equivalente a las actividades físicas anteriormente mencionadas.

En cuanto al comportamiento sedentario, se definió como el tiempo excesivo frente a una pantalla, de dos o más horas diarias de tiempo no relacionado con el trabajo, como utilización del televisor, computador, juegos de video, tabletas o celular. La ENSIN 2015 utilizó, para la recolección de los datos de actividad física y de conductas sedentarias, la versión larga del cuestionario internacional de actividad física *(International Physical Activity Questionnaire,* IPAQ) ^16^.

Además, en este estudio, se consideró el análisis por variables, el cual incluyó sexo, edad, área geográfica, región, índice de riqueza y etnia (para esta última, la categoría afrodescendiente incluye negro, afrocolombiano, mulato y palenquero de San Basilio).

Para la recolección de la información, se diseñó y ejecutó un sistema de captura, con una malla de validación con aspectos de control para la correcta aplicación de la metodología de la ENSIN. El sistema aplicaba las reglas de selección para cada componente, según el grupo etario y el sexo, lo que permitió contar con el estado de aplicación hasta la unidad final de observación de manera unificada y centralizada. Se realizaban cruces preliminares, con el propósito de hacer una evaluación de la validez de los datos e identificar posibles defectos.

Los datos también fueron revisados técnicamente por los críticos y depuradores, a fin de identificar, según la temática, posibles errores, inconsistencias o datos atípicos, lo cual permitió, no solamente la mejoría de los procedimientos de recolección en campo, sino la completitud de la información. Como respaldo al proceso de recolección de información y garantía de la unicidad de los criterios, se contó con manuales de procesos y procedimientos para cada rol, formatos de control diario, y manual de crítica, validación y consistencia.

Para analizar la información, se empleó el *software* Stata 12, además del comando *svy* para muestras complejas de datos provenientes de encuestas ^17^, a partir del cual se estimaron proporciones e intervalos de confianza al 95 %, ajustados por el diseño de la encuesta. Para las comparaciones estadísticas de las variables categóricas, se empleó el valor estadístico de ji al cuadrado y, para evaluar la diferencia entre las categorías de las variables, se ejecutó el comando *lincom* en Stata, que calcula el valor de p de coeficientes después de la estimación para encuestas *(svy).* Se utilizó una significancia de 0,05 en las pruebas de hipótesis ^17^.

Se construyeron modelos de regresión logística binaria, en los cuales se estudiaba la asociación entre la prevalencia de obesidad y las variables sociodemográficas, inicialmente regresiones logísticas simples entre la variable dependiente (obesidad) y cada una de las variables independientes; posteriormente, se construyó un modelo multivariado, con las variables que en las regresiones logísticas simples tuvieran al menos una de sus categorías significativas (p≤0,05).

Para analizar la probabilidad de ocurrencia del evento, se utilizó la significancia con la razón de momios *(odds ratio,* OR): una OR de 1 implicó sin diferencia entre las categorías comparadas, una OR mayor de 1, una asociación positiva entre las variables, y una OR menor de 1, una asociación negativa.

Esta investigación se llevó a cabo a partir de un análisis a profundidad de la base de datos anonimizados de una encuesta poblacional, y de acceso público en el Repositorio Institucional Digital del Ministerio de Salud y Protección Social, por tanto, se cumplieron con las consideraciones éticas internacionales ^18^ y vigentes en Colombia ^19^, incluyendo el consentimiento informado de los padres y el aval del Comité de Ética del Instituto Nacional de Salud según Acta no. 2-2015 del 16 de febrero de 2015.

## Resultados

Del total de la población analizada (16.607), el 54,8 % eran mujeres y el 45,2 % eran hombres. El 88,5 % se autorreconoció como sin pertenencia étnica, el 77,6 % vivía en viviendas ubicadas en el área urbana, una cuarta parte pertenecía a la región central y el 50,6 % estaba afiliado al régimen contributivo del Sistema General de Seguridad Social en Salud. En relación con la escolaridad y la actividad económica principal, el 47,6 % tenía secundaria completa y el 54,3 % reportó trabajo informal (cuadro 1).

**Cuadro 1:** Características sociodemográficas de la población de 18 a 64 años

Variables	n^a^	%	IC^b^	
Sexo				
Hombres	7.936	45,2	43,7	46,8
Mujeres	8.671	54,8	53,2	56,3
Edad (años)				
18 a 29	5.159	33,6	32,4	34,7
30 a 49	6.930	42,9	41,4	44,4
50 a 64	4.509	23,5	22,2	24,9
Etnia				
Negro-afro^c^	1.467	7,6	6,6	8,8
Indígena	1.346	3,9	2,8	5,3
Sin pertenencia étnica	13.657	88,5	87	89,9
Área				
Urbana	12.360	77,6	75,4	79,6
Rural	4.247	22,4	20,4	24,6
Región				
Atlántica	3.555	22,6	20,4	24,8
Oriental	2.891	17,3	14,3	20,6
Orinoquía y Amazonía	2.647	2,3	2	2,7
Bogotá	1.231	16,8	14,1	19,7
Central	3.517	25,1	23,2	27,1
Pacífico	2.766	16	14,6	17,5
Nivel de escolaridad				
Primaria incompleta	3.244	15,4	14,2	16,6
Primaria completa	5.127	28,2	27	29,4
Secundaria completa	6.999	47,6	46	49,2
Superior	1.158	8,9	7,7	10,2
Actividad económica				
Desempleado	499	5	4,3	5,8
Trabajo informal	4.655	54,3	51,6	57
Trabajo formal	5.356	40,7	38,1	43,2
Aseguramiento en salud				
Régimen contributivo	6.621	50,6	48,3	52,9
Régimen subsidiado	8.989	43,6	41,4	45,8
No afiliado	924	5,8	5,1	6,6
Recomendación de actividad física				
de 150 minutos por semana				
No cumple	8.097	48,3	46,8	49,9
Cumple	8.501	51,7	50,1	53,2
Tiempo excesivo frente a pantalla				
2 horas/día	8.926	56,9	55,4	58,4
0-1,99 horas/día	7.672	43,1	41,6	44,6

^a^ Se incluyeron solo personas residentes habituales. Número de individuos sin ponderar. Los porcentajes tienen en cuenta la ponderación y las etapas de diseño.

^b^ Intervalo de confianza del 95 %

^c^ Integra negro, afrocolombiano, afrodescendiente, mulato y palenquero de San Basilio

Como se muestra en el cuadro 2, más de la mitad (55,9 %) de los adultos participantes presentaba exceso de peso; la proporción de personas con sobrepeso fue el doble (37,1 %) comparada con los que presentaron obesidad (18,8 %).

**Cuadro 2:** Estado nutricional por IMC de la población de 18 a 64 años

IMC	n^a^	%^b^	IC^c^	
Delgadez	376	2,5	2,1	2,9
Normal	6.733	41,6	40,4	42,8
Exceso	9.498	55,9	54,7	57,1
Sobrepeso	6.170	37,1	35,8	38,4
Obesidad	3.328	18,8	17,7	19,8

^a^ Número de individuos sin ponderar

^b^ Los porcentajes tienen en cuenta la ponderación y las etapas de diseño.

^c^ Intervalo de confianza del 95 %

Al analizar la desagregación por variables sociodemográficas de los participantes según el estado nutricional por IMC (cuadro 3), se encontró que los hombres tenían más sobrepeso (37,6 %) que las mujeres, mientras que en estas fue más frecuente la obesidad (22,1 %). Las mayores prevalencias de sobrepeso y obesidad fueron halladas en los rangos de 30 a 49 años (41,9 % sobrepeso y 21,6 % obesidad) y de 50 a 64 años (43,9 sobrepeso y 24,8 obesidad). En el grupo de 18 a 29 años, se encontraron los menores porcentajes de sobrepeso (26,4 %) y obesidad (10,7 %) y las mayores prevalencias de delgadez (4,6 %).

**Cuadro 3:** Variables sociodemográficas según IMC de la población de 18 a 64 años

Variables	Delgadez		Normal	Exceso	Sobrepeso	Obesidad
	n^a^	%^b^	%^b^	%^b^	%^b^	%^b^
Sexo						
Hombres	7.936	2,9	44,9	52,2	37,6	14,6
Mujeres	8.671	2,2	38,9	58,9	36,8	22,1
Edad (años)						
18 a 29	5.159	4,6	58,3	37,1	26,4	10,7
30 a 49	6.930	1,5	35	63,5	41,9	21,6
50 a 64	4.509	1,3	30	68,7	43,9	24,8
Etnia						
Afrodescendiente	1.467	2,1	41	56,9	31	25,9
Indígena	1.346	1,2	49,5	49,3	35,5	13,8
Sin pertenencia étnica	13.657	2,6	41,3	56,1	37,7	18,4
Área						
Urbana	12.360	2,6	40,2	57,2	37,5	19,7
Rural	4.247	2,1	46,6	51,3	35,9	15,4
Región						
Atlántica	3.555	3,9	40,3	55,8	35,3	20,5
Oriental	2.891	2	44,8	53,2	36,8	16,4
Orinoquía y Amazonía	2.647	1,9	40,3	57,8	37,1	20,7
Bogotá	1.231	2,4	45,2	52,4	37,1	15,3
Central	3.517	2	40,2	57,8	38,5	19,3
Pacífico	2.766	2	38,9	59,1	38	21,1
Cuartil de riqueza						
Muy bajo	7.371	2,9	46,1	51	35	16
Bajo	3.943	3,1	41,2	55,7	35,5	20,2
Medio	3.123	2,2	38,8	59	38,4	20,6
Alto	2.170	1,6	39,3	59,1	40,1	19
Nivel de escolaridad						
Primaria incompleta	3.244	2,4	39,5	58,1	37,1	21,0
Primaria completa	5.127	2,3	39,9	57,7	37,0	20,8
Secundaria completa	6.999	2,8	44,1	53,1	36,0	17,1
Superior	1.158	1,5	37,6	60,9	43,6	17,4
Actividad económica						
Desempleado	499	2,8	46,4	50,7	40,2	10,5
Trabajo informal	4.655	1,4	39,5	59,1	41,1	18,0
Trabajo formal	5.356	2,4	44,3	53,3	35,8	17,5
Aseguramiento en salud						
Régimen contributivo	6.621	1,6	39,9	58,5	39,8	18,7
Régimen subsidiado	8.989	3,3	43,2	53,5	34,3	19,2
No afiliado	924	3,7	44,9	51,3	35,9	15,4

^a^ Se incluyeron solo personas residentes habituales. Número de individuos sin ponderar.

^b^ Los porcentajes tienen en cuenta la ponderación y las etapas de diseño.

La mayor proporción de personas con obesidad se presentó en los participantes de etnia afrodescendiente (25,9 en la zona urbana (19,7 %), en el cuartil medio (20,6 %) y el bajo (20,2 %) de riqueza, en la región Pacífica (21,1 %) seguida por la región Orinoquía y Amazonía (20,7 %) y la región Atlántica (20,5 %), en el nivel de escolaridad primaria incompleto (21 %), actividad económica informal (18 %) y en el régimen subsidiado (19,2 %) de aseguramiento en salud.

Analizando si se cumple o no la recomendación de realizar 150 minutos de actividad física en la semana, incluyendo el transporte, se encontró que el 48,3 % no cumple la recomendación; es importante destacar que el 56,9 % de los adultos pasan dos o más horas diarias frente a pantallas, considerándose esto como un comportamiento sedentario (cuadro 4).

**Cuadro 4:** Recomendación de actividad física y tiempo frente a pantalla

Variable	n	%	IC	
Recomendación de actividad física de 150 minutos por semana				
No cumple	8.097	48,3	46,8	49,9
Cumple	8.501	51,7	50,1	53,2
Tiempo en pantalla				
≥2 horas/día	8.926	56,9	55,4	58,4
0-1,99 horas/día	7.672	43,1	41,6	44,6

Los porcentajes tienen en cuenta la ponderación y las etapas de diseño.

Al analizar la relación entre actividad física, tiempo en pantalla y estado nutricional por análisis bivariado, se encontró una significación estadística (p<0,05) entre no cumplir con las recomendaciones de actividad física y la obesidad en la población adulta, sin diferencias por rangos de edad. La asociación entre tiempo excesivo ante la pantalla y estado nutricional solamente fue significativa en los adultos que presentaron obesidad en edades entre los 50 y los 64 años (p=0,015). No se encontró relación entre cumplir la recomendación de actividad física y el tiempo frente a pantalla.

En la desagregación por variables sociodemográficas, el resultado obtenido es una significancia entre obesidad y sexo: las mujeres tuvieron una p<0,000 en relación con los hombres. También, se hallaron diferencias significativas relacionadas con el área de residencia: quienes vivían en el área urbana (p<0,000), en la región pacífica (p<0,005), los pertenecientes a la etnia afrodescendiente (p<0,000) y al cuartil medio de riqueza (p<0,002).

En cuanto a la actividad física por sexo, son los hombres quienes cumplen con la recomendación con una diferencia significativa de p<0,000, así mismo, los que viven en la región oriental (p<0,001) y los indígenas (p<0,006). Sin embargo, no se presentaron diferencias significativas entre los adultos que viven en área urbana o rural y el cuartil de riqueza. El tiempo excesivo frente a la pantalla se relacionó con el área urbana (p<0,000), vivir en la región Bogotá (p<0,000), ser afrodescendiente (p<0,000) y pertenecer al cuartil de riqueza alto (p<0,011). Por otra parte, no se presentó diferencia significativa entre el sexo masculino y el femenino.

De los análisis de los modelos de regresión, se seleccionaron las variables en las que, por lo menos, una de sus categorías fuera significativa y, con estas, se definió un modelo multivariado con nueve variables y se construyó un modelo resumido solo con las variables significativas del anterior (cuadro 5), considerado el más parsimonioso y sobre el cual se destaca que los adultos entre los 30 y los 49 años (OR=3,05; IC_95%_: 2,673,48) y entre los 50 y los 64 años (OR=4,02; IC_95%_: 3,44-4,69) de edad, tienen más probabilidades (entre 3 y 4 veces, respectivamente) de presentar obesidad que los más jóvenes (18 a 29 años).

**Cuadro 5:** Análisis de regresión logística en población adulta

Variables	Regresiones bivariadas				Modelo multivariado 1				Modelo multivaiado final			
	OR^a^	IC_95%_^b^		p^c^	OR	IC_95%_		p	OR	IC_95%_		p
Sexo												
Hombres												
Mujeres	1,31	1,18	1,46	0,00	1,07	0,92	1,24	0,40				
Edad (años)												
18 a 29												
30 a 49	2,94	2,59	3,33	0,00	2,72	2,33	3,17	0,00	3,05	2,67	3,48	0,00
50 a 64	3,71	3,21	4,30	0,00	3,19	2,63	3,86	0,00	4,02	3,44	4,69	0,00
Etnia												
Sin pertenencia étnica												
Negro-afro^c^	1,03	0,88	1,21	0,72	0,99	0,77	1,26	0,91				
Indígena	0,76	0,61	0,95	0,02	1,19	0,88	1,59	0,26				
Área												
Rural												
Urbana	1,27	1,15	1,40	0,00	1,29	1,11	1,51	0,00	1,28	1,15	1,44	0,00
Región												
Bogotá												
Atlántica	1,15	0,95	1,40	0,16	1,59	1,23	2,04	0,00	1,41	1,15	1,73	0,00
Oriental	1,03	0,85	1,27	0,71	1,28	0,97	1,68	0,08	1,24	1,01	1,52	0,04
Orinoquía y Amazonía	1,25	0,98	1,59	0,08	2,03	1,53	2,66	0,00	1,56	1,25	1,95	0,00
Central	1,25	1,03	1,52	0,03	1,48	1,15	1,89	0,03	1,40	1,15	1,71	0,00
Pacífico	1,32	1,07	1,61	0,01	1,72	1,30	2,27	0,00	1,58	1,28	1,95	0,00
Nivel de escolaridad												
Primaria incompleta												
Primaria completa	0,98	0,85	1,14	0,83	1,35	1,13	1,61	0,00	1,22	1,02	1,41	0,01
Secundaria completa	0,82	0,71	0,93	0,00	1,39	1,14	1,69	0,00	1,26	1,08	1,47	0,00
Superior	1,12	0,88	1,44	0,35	1,25	0,90	1,74	0,18	1,26	0,98	1,63	0,07
Actividad económica												
Desempleado												
Trabajo informal	1,40	1,07	1,84	0,02	0,92	0,69	1,22	0,55				
Trabajo formal	1,11	0,84	1,45	0,46	1,13	0,58	2,18	0,72				
Aseguramiento en salud												
No afiliado												
Régimen contributivo	1,33	1,04	1,70	0,02	0,96	0,46	2,00	0,92				
Régimen subsidiado	1,09	0,86	1,39	0,47	0,96	0,70	1,31	0,81				
Recomendación actividad física												
de 150 minutos por semana												
No cumple												
Cumple	0,76	0,68	0,84	0,00	0,78	0,68	0,89	0,00	0,80	0,72	0,89	0,00
Tiempo excesivo frente a pantalla												
2 horas/día	1,01	0,91	1,12	0,811								

^a^ *Odds ratio*

^b^ Intervalo de confianza del 95 %

^c^ valor de p

Se presentó una probabilidad positiva con vivir en el área urbana (OR=1,28; IC_95%_: 1,15-1,44) en comparación con el área de referencia (rural); lo mismo sucede con haber completado la secundaria (OR=1,26; IC_95%_: 1,08-1,47) y tener un nivel educativo menor de éste, además de vivir en el área urbana (OR=1,28; IC_95%_: 1,15-1,44) y en una región diferente a Bogotá p<0,05. Por otra parte, es importante resaltar que cumplir la recomendación de actividad física disminuye 1,25 veces la probabilidad de tener obesidad.

## Discusión

En Colombia, casi el 50 % de los adultos presenta inactividad física, lo que representa el doble en comparación con las cifras mundiales; se encontró que uno de cada cuatro adultos no cumple con las recomendaciones de actividad física establecidas por la OMS ^3^, en relación con los cambios en los patrones de transporte, una mayor utilización de la tecnología y la urbanización.

Nuestros resultados, al igual que los de otros trabajos ^20^^,^^21^, muestran desigualdad por sexo frente al cumplimiento de la recomendación de actividad física, pues es mayor el porcentaje de hombres que la cumplen. Esto podría estar relacionado con los datos reportados por el Departamento Administrativo Nacional de Estadística (DANE) de los años 2020 y 2021 a nivel nacional: las mujeres destinaron 7 horas 0 minutos al día en promedio a las actividades de trabajo no remunerado, en tanto que los hombres solo lo hicieron durante 3 horas 6 minutos ^22^. Además, con el reporte de 2021, en que se demuestra que las mujeres proporcionaron el 77,7 % del total de las horas al año en la realización de trabajo doméstico y de cuidado no remunerado, mientras que los hombres aportaron solo el 22,3 %, lo cual puede disminuir su oportunidad de realizar actividad física ^23^. Además, se demostró la asociación entre el incumplimiento de la actividad física y ser obeso, lo que es consistente con las mayores prevalencias encontradas, tanto de obesidad como de inactividad física en las mujeres.

Aun cuando no se presentaron diferencias significativas por edad en relación con el cumplimiento de las recomendaciones de actividad física, la mayor proporción de obesidad se encontró en adultos entre los 30 y los 64 años, valor que duplica el encontrado entre los menores de 30 años. Esto podría estar relacionado, como lo indica la información del Ministerio de Salud y Protección Social a 2021 ^24^, con la mayor frecuencia de enfermedades o lesiones osteomusculares en personas mayores de 40 años, que limitan la realización de actividad física o disminuyen su intensidad. En otros estudios, como el de Martins *et al.,* sí se encontraron diferencias significativas por edad relacionadas con la práctica de actividad física (p<0,007) ^25^.

Por otra parte, se ha incrementado la evidencia por medio de datos prospectivos de que el comportamiento sedentario puede estar relacionado con el riesgo de desarrollar algunas enfermedades crónicas no transmisibles (diabetes mellitus y enfermedad cardiovascular), así como con la mortalidad general ^26^^,^^27^. En este sentido, en múltiples estudios se han medido los comportamientos sedentarios, tanto por autorreporte de los entrevistados como con la utilización de herramientas de medición, y se ha encontrado asociación entre la raza o etnicidad y el comportamiento sedentario en muestras grandes de adultos. La mayoría se ha centrado en el tiempo de visualización de la televisión, encontrándose comúnmente que los de raza negra ven más televisión que los adultos de otras etnias ^28^^,^^29^. Lo mismo ocurrió en esta investigación, donde se presentó una asociación entre el tiempo excesivo frente a pantalla mayor o igual a dos horas y ser afrodescendiente.

Asimismo, es importante resaltar que en este estudio no se encontró asociación entre el tiempo excesivo en pantalla de más de dos horas y la probabilidad de tener obesidad; sin embargo, el cumplir con la recomendación de realizar actividad física disminuye la probabilidad de estar obeso. Otros estudios, como el de Shin ^30^, presentan una asociación significativa entre la obesidad y pasar principalmente más de seis horas al día frente a una pantalla, sin asociaciones significativas entre la actividad física y la obesidad. Estos resultados pueden deberse a la diferencia en el tiempo de referencia de la medición frente a pantalla.

En Colombia, la obesidad se considera un problema de salud pública en la población adulta, con mayor prevalencia de las mujeres y los adultos mayores de 30 años, que se relaciona con no cumplir la recomendación de actividad física. Por lo anterior, es importante practicar más actividad física moderada y vigorosa para reducir la prevalencia de obesidad, especialmente, en el caso de las personas sedentarias.

El análisis del comportamiento sedentario, medido por tiempo excesivo frente a pantallas, mostró mayores prevalencias en los afrodescendientes, personas pertenecientes al cuartil alto de riqueza y los que residían en el área urbana y en la región Bogotá.
